# Acute Generalized Exanthematous Pustulosis due to Tocilizumab in a Rheumatoid Arthritis Patient

**DOI:** 10.1155/2012/517424

**Published:** 2012-12-12

**Authors:** J. H. Izquierdo, F. Bonilla-Abadía, C. D. Ochoa, A. Agualimpia, G. J. Tobón, C. A. Cañas

**Affiliations:** Rheumatology Unit, Valle del Lili Foundation, ICESI University, Cali, Colombia

## Abstract

We report a female patient with rheumatoid arthritis which was refractory to methotrexate, leflunomide, and anti-TNF therapy. She was treated with anti-IL-6 tocilizumab (TCZ), with an early appearance of sterile pustules on erythematous swollen skin of trunk, back, and abdominal area. The lesions were consistent with the diagnosis of acute drug-related generalized exanthematous pustulosis (AGEP). This adverse event was controlled with medical treatment without requiring removal of TCZ.

## 1. Introduction

Rheumatoid arthritis (RA) is a common autoimmune disease of unknown cause associated with progressive disability and diverse systemic complications [1]. The early use of traditional disease-modifying antirheumatic drugs (DMARD) in a “treatment-to-target” strategy, currently have improved the prognosis. However, there are patients who do not achieve the goal of disease remission. Patients that have an inadequate response to traditional DMARD, including methotrexate (MTX), have been benefited of antitumor necrosis factor *α* (anti-TNF*α*) agents. However, 20–40% of these patients do not respond well to anti-TNF*α* therapy [2]. Tocilizumab (TCZ), an anti-interleukin-6 therapy, is approved for the treatment of moderate to severe rheumatoid arthritis including patients with anti-TNF*α*-therapy failure [3]. Different adverse events associated to TCZ have been reported, including infections, neutropenia hepatic toxicity, and bowel complications [4]. There were no reports found of drug-related rash or exanthematous eruptions with TCZ in the medical literature. We report a case of a patient with refractory RA, requiring the use of TCZ and who presented an acute generalized exanthematous pustulosis (AGEP).

## 2. Case Report

**Figure 1:**
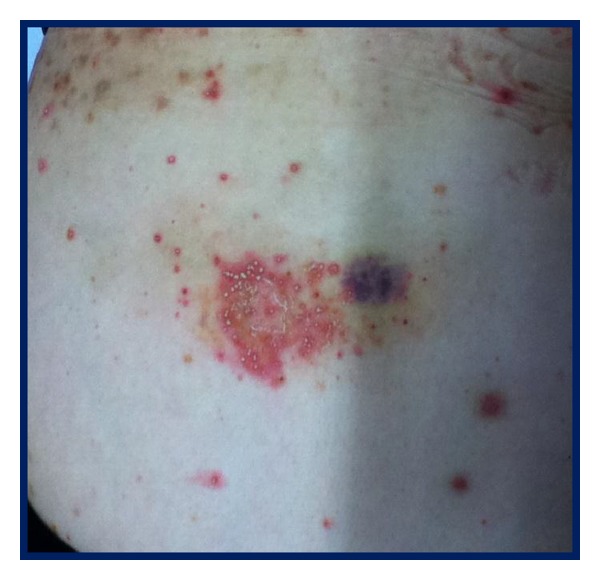
AGEP TCZ related lesions in reported patient with refractory RA.

A 46-year-old female patient diagnosed with RA for two years was remitted because of a failure of disease control with conventional management. She showed persistence of arthritis, mainly in metacarpophalangeal and proximal interphalangeal joints, associated with morning stiffness and functional disability. Physical examination showed synovitis in metacarpophalangeal joints and wrists. Their laboratory test showed a C-reactive protein (CRP) 1.2 mg/dL, erythrocyte sedimentation rate of 105 mm/h, hemoglobin: 11.6 g/dL, creatinine: 0.76 mg/dL, AST: 49 U/l, and ALT: 32 U/l. Radiographic erosions in metacarpophalangeal and proximal interphalangeal joints were evidenced. Initially she received prednisolone 5 mg/day, hidroxychloroquine 250 mg/day, and MTX 10 mg weekly with progressive increase to 20 mg/week without response and requiring combination with leflunomide 20 mg/day for 3 months with persistence of synovitis and elevated acute phase reactants. Etanercept 50 mg/weekly was began with partial improvement of symptoms. Three months later she suffered from pain in fingers, wrists, and knees, with inflammatory signs in metacarpophalangeal joints. Then etanercept was replaced by TCZ (8 mg/kg monthly). Two weeks later of the first infusion, she consulted due to the presence of cutaneous rash in trunk, back, and abdominal area (Figure 1) without fever. The lesions were sterile nonfollicular pustules on erythematous swollen skin. The blood cell count reported mild neutrophilia without eosinophilia. A diagnosis of AGEP was done. We continued therapy with TCZ, increased temporally prednisolone dose to 50 mg/day, and began topical glucocorticoid, achieving, resolution of cutaneous condition; therefore no biopsy was performed. Four months later, the patient presented remission of RA and AGEP, with reduction of prednisolone dose to 5 mg/day and normal levels for acute-phase reactants.

## 3. Discussion

Remarkable changes have been reported following the introduction of biological therapies in RA; such treatments are now firmly established in rheumatological practice [5]. Several studies support the role of TCZ in the control of clinical and radiological progression of RA [6]. The data from clinical trials lead to the approval of TCZ for the treatment of moderate to severe RA, in combination with MTX, in patients who had inadequate response to one or more anti-TNF drugs [7]. Safety concerns are similar to other biologic DMARDs, especially infections and rare infusion-related reactions. Other adverse events include decrease in neutrophils, increase of liver enzymes and, low and high density lipoproteins. There are also some reports about gastrointestinal perforation, and caution must be used in patients with previous diverticulosis [8]. However, there are no reports founded about drug-related rash in the literature with the use of TCZ. Our patient presented AGEP, an exanthematous drug eruption, two weeks after the onset of TCZ. This cutaneous condition has a benign presentation with rapid resolution, rare mucosal involvement, fever (>38.5°C), and leukocytosis with neutrophilia. Typical cases with drug-related AGEP do not require histological confirmation [9]. Our patient has been clinically diagnosed and treated.

We reported a case of typical AGEP related to TCZ in a patient with RA. The patient presented resolution from cutaneous lesions with short oral cycle and topic glucocorticoids, without the need of TCZ withdrawal.
